# “*Laro, Layunin, Lagablab*”: exploring study engagement in Filipino recreational games-based physical education through the tripartite model of individual interest

**DOI:** 10.3389/fspor.2026.1797819

**Published:** 2026-06-02

**Authors:** Joseph Lobo

**Affiliations:** 1 College of Sports, Exercise and Recreation, Bulacan State University, Malolos, Philippines; 2 Graduate School, Bulacan State University, Malolos, Philippines; 3 INTI International University, Nilai, Malaysia

**Keywords:** higher education, individual interest, physical education, recreational games, study engagement

## Abstract

**Introduction:**

Culturally grounded physical education provides meaningful opportunities to connect movement, heritage, and student engagement. This study examined the association between individual interest and study engagement in Filipino recreational games-based physical education among university students in the Philippines. Filipino recreational games, locally known as *laro ng lahi*, are traditional movement-based activities commonly integrated into PE to promote cultural appreciation and active participation. Guided by the tripartite model, individual interest was conceptualized through three factors: positive affect and willingness to reengage (PAWR), stored-utility value (SUV), and stored attainment value and knowledge-seeking intentions (SAVKSI).

**Methods:**

A total of 361 valid responses were analyzed using hierarchical multiple regression analysis.

**Results:**

Results indicated that individual interest was significantly associated with students' study engagement and accounted for a substantial proportion of its variance. All three components were significantly associated with study engagement, with SAVKSI demonstrating the strongest association, highlighting the role of internalized goals and knowledge-oriented motivations in sustaining engagement in physical education.

**Discussion:**

These findings support the applicability of the tripartite model within culturally grounded PE contexts and emphasize the importance of designing meaningful and culturally relevant movement experiences. Implications for curriculum development, student motivation, and pedagogical design in movement-based education are discussed.

## Introduction

As digital entertainment, academic competition, and technology-mediated lifestyles increasingly shape the lived experiences of Filipino university students (1–3), Physical Education (PE) finds itself at a pedagogical crossroads. Although historically positioned as a vital component of holistic education integrating physical, social, emotional, and moral development (4, 5), PE has increasingly been marginalized within higher education (6, 7). In many Philippine universities, it is often reduced to a compliance-based requirement with limited instructional innovation and student regard (8, 9). This marginalization is reflected in declining participation and limited affective engagement among students, alongside the growing normalization of sedentary lifestyles (10, 11). These conditions highlight the need to reposition PE as a meaningful and engaging component of university education (12).

In the Philippine context, PE is implemented across educational levels as part of the national curriculum promoting physical literacy, health and lifelong physical activity (13, 14). At the basic education level, PE is delivered through the Music, Arts, Physical Education and Health (MAPEH) learning area, while Senior High School (SHS) adopts a more specialized approach through subjects such as Health Optimizing Physical Education (HOPE) (15, 16). At the tertiary level, students typically complete a sequence of Physical Activity Towards Health and Fitness (PATH-Fit) courses, exposing them to diverse movement forms including sports, dance, fitness activities, and culturally rooted games (14). Despite this structured implementation, PE is frequently perceived as peripheral in higher education, which may contribute to reduced student engagement (17, 18).

**Figure 1:**
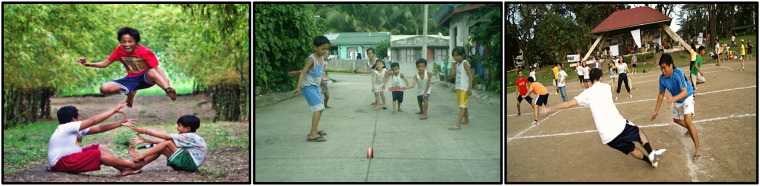
Examples of Filipino recreational games commonly incorporated in physical education contexts. From left to right: *Luksong tinik*, a cooperative jumping game where players leap over progressively higher obstacles formed by participants’ hands and feet; *tumbang preso*, a chasing and target game where players attempt to knock down a standing object using a slipper while avoiding being tagged; and *patintero*, a team-based grid tag game requiring players to evade defenders while crossing designated lines. These images are included for illustrative purposes only and do not depict participants from the present study. Patintero image © Yabang Pinoy (CC BY 2.0), Wikimedia Commons. Luksong tinik image © Erick John Cuevas (CC BY-SA 4.0), Wikimedia Commons. Tumbang preso image © Government of the Philippines (Public Domain—PD-Philippines), Wikimedia Commons.

A culturally grounded resource with strong pedagogical potential is Filipino recreational games or laro ng lahi (19). These constitute a vibrant collection of traditional Filipino games such as *patintero*, a team-based grid tag game requiring players to evade defenders while crossing designated lines; *luksong tinik*, a cooperative jumping game in which players leap over progressively higher obstacles formed by participants' hands and feet; and *tumbang preso*, a chasing game where players attempt to knock down a standing object (commonly a can) using a slipper while avoiding being tagged (see Figure 1). These traditional games embody cooperation, agility, resilience and community participation (20). Beyond their role in cultural preservation, they represent forms of embodied learning that integrate physical, emotional and social dimensions (21). However, despite their relevance, these games remain underutilized in higher education PE curricula (19). Empirical research examining their role in shaping academic constructs such as motivation and engagement remains limited (22, 23), highlighting a critical gap in culturally responsive PE research.

### Cultural and pedagogical significance of Filipino recreational games in physical education

Filipino recreational games, or “*laro ng lahi*”, are an integral component of the nation's intangible cultural heritage (21). These traditional games emerged organically from indigenous play culture and were historically embedded in community life (24). Beyond leisure, they function as socially constructed activities that promote cooperation, creativity, agility, and problem-solving (25, 26). They also cultivate culturally grounded values such as *bayanihan* (communal unity), *diskarte* (practical ingenuity) and *pakikipagkapwa-tao* (shared humanity), aligning closely with the holistic aims of Physical Education (95).

Although traditionally associated with childhood, these games hold pedagogical relevance across age groups. Their cooperative and open-ended nature fosters inclusive, low-resource and socially interactive learning environments that support movement literacy and character development (19, 27). Unlike performance-oriented PE models that emphasize competition and standardization, *laro ng lahi* adopt a play-based paradigm that encourages participation, agency, and cultural immersion (28). These characteristics make them particularly suited for fostering sustained engagement in movement-based learning.

Despite these advantages, Filipino recreational games remain underutilized in contemporary higher education PE settings (29). The increasing dominance of structured sports and fitness-oriented curricula, often influenced by Western models, has contributed to the marginalization of indigenous movement practices (30, 31). This shift limits students' exposure to culturally resonant and community-rooted forms of physical activity, which may otherwise support meaningful engagement.

(Re)positioning *laro ng lahi* within university PE aligns with calls for culturally sustaining pedagogies that recognize students' heritage as a valuable resource for learning (32). In this context, these games offer a platform where cultural identity, community participation, and embodied learning intersect (20, 33). However, despite their pedagogical promise, limited empirical evidence exists on how such culturally grounded practices influence academic constructs such as motivation and engagement among university students. This gap underscores the need to examine their role not only as cultural artifacts but as pedagogical tools that can support meaningful engagement in Physical Education.

### Individual interest as a motivational construct in physical education

*Individual interest* is a central construct in educational psychology, particularly in understanding motivation and engagement (34). It is defined as a relatively enduring predisposition to reengage with specific content over time (35). Unlike situational interest, which is temporary and externally triggered, individual interest reflects internalized motivation and personal relevance developed through repeated meaningful experiences (36). Its development is shaped by sustained exposure, perceived value and social support, making it highly applicable in movement-based contexts such as Physical Education (37).

The Four-Phase Model of Interest Development (37) explains how learners progress from externally triggered curiosity to well-developed individual interest. Within this framework, sustained interest emerges when learners encounter personally meaningful content, experience positive affect and are provided opportunities for active engagement. In PE, this highlights the importance of designing learning environments that foster both emotional and cognitive involvement, conditions that may be supported by culturally grounded practices such as *laro ng lahi*.

To operationalize individual interest in this study, the Tripartite Model of Individual Interest (38) was adopted. This model conceptualizes interest through three interrelated factors: *positive affect and willingness to reengage* (PAWR), *stored-utility value* (SUV), and *stored attainment value and knowledge-seeking intentions* (SAVKSI). These factors capture both affective and value-based aspects of sustained interest and provide a structural explanation of how interest contributes to engagement in learning contexts (38).

The model is theoretically anchored in Self-Determination Theory (38) and Expectancy-Value Theory (40). From an SDT perspective, PAWR reflects intrinsic enjoyment and autonomy in reengaging with an activity, while SAVKSI aligns with competence and intrinsic motivation through its emphasis on personal growth and knowledge-seeking (41, 42). SUV corresponds to the utility value component of EVT, representing the perceived usefulness of an activity in achieving future goals (43). Together, these factors explain how students develop enduring interest not only through enjoyment, but also through perceived relevance and personal meaning.

Empirical applications of the tripartite model in Philippine higher education have demonstrated its utility across various Physical Education contexts. Studies conducted in general PE, gymnastics and Philippine folk dance consistently show that individual interest is positively associated with student engagement (44–46). These findings highlight the context-sensitive nature of interest, suggesting that its role may vary depending on how PE activities are designed and experienced. However, despite this growing body of evidence, the model has not been applied to Filipino recreational games. This represents a critical gap in understanding how culturally grounded movement practices are associated with motivation and engagement in Physical Education. Although prior studies have applied the tripartite model of individual interest across various physical education contexts, including culturally grounded domains, the present study extends this line of inquiry by examining its applicability within Filipino recreational games (*laro ng lahi*). In doing so, the study contributes to understanding the cross-context applicability and robustness of the model in diverse movement-based learning environments.

### Study engagement in physical education and its theoretical underpinnings

As Physical Education continues to evolve in response to the demands of contemporary learners, student engagement has emerged as a key outcome in both pedagogical research and curriculum design (47). In PE contexts, engagement extends beyond physical participation to include students' cognitive investment, emotional involvement and behavioral effort in learning activities (48, 49). Engaged students are not only physically active but also meaningfully involved in interpreting movement experiences and connecting them to personal and cultural contexts (50).

*Study engagement* is commonly conceptualized within Work Engagement Theory (51), which defines it as a positive, fulfilling state of mind characterized by vigor, dedication and absorption (52, 53). These dimensions represent students' energy, commitment and immersion in academic tasks. In this study, engagement was measured using the Utrecht Work Engagement Scale for Students (UWES-9S), a validated instrument widely applied in educational and sport-related contexts (54, 55).

Within PE, these dimensions are reflected in students' willingness to exert effort, their emotional investment in movement tasks and their capacity to remain focused during embodied learning activities (56). Higher levels of engagement are associated with more meaningful learning experiences, including greater receptiveness to feedback, collaboration with peers, and persistence when facing challenges (57). Engagement in PE has also been linked to broader educational outcomes, such as improved self-efficacy and more positive attitudes toward physical activity beyond the classroom (58).

Student engagement is shaped by a combination of personal, contextual and pedagogical factors, with motivation consistently identified as a key contributor (58). When students perceive PE activities as relevant, meaningful and emotionally resonant, they are more likely to invest cognitive and affective resources in learning (59). However, despite growing interest in engagement research, its role within culturally grounded movement contexts such as *laro ng lahi* remains underexplored. This highlights the need to examine how engagement operates within such contexts and how it relates to motivational constructs like individual interest.

### Empirical evidence linking individual interest and study engagement in physical education

Empirical research within Philippine higher education consistently supports the role of individual interest in relation to study engagement across various Physical Education contexts. These studies show that students' affective experiences, perceived utility and value-oriented motivations toward PE activities are associated with their level of engagement. For instance, a large-scale study involving 11,378 college students enrolled in general PE found that all three dimensions of individual interest were significantly associated with study engagement, with stored attainment value and knowledge-seeking intentions (SAVKSI) emerging as the strongest factor (45). A similar pattern was observed in subsequent work examining PE participation across institutions, where individual interest dimensions remained significantly associated with engagement outcomes (60).

Variations in these associations have also been observed across different PE contexts. In gymnastics-based PE, only stored-utility value (SUV) was significantly associated with engagement, highlighting the relevance of perceived functional benefits in more performance-oriented activities (46). In contrast, in culturally grounded contexts such as Philippine folk dance, all three dimensions were significantly associated with engagement, with SAVKSI again emerging as the most prominent factor (44). These findings suggest that the role of individual interest may differ depending on the nature and context of the movement activity.

These local findings align with broader international research indicating that individual interest is positively associated with academic engagement and persistence (61, 62). Interest has been described as both a motivational driver and an affective filter that supports sustained attention and commitment in learning (63). Although much of this work has been conducted in classroom-based settings, its implications extend to movement-based learning contexts where cognitive and affective engagement are equally relevant.

Further evidence from diverse contexts reinforces the importance of individual interest in Physical Education. Studies have shown that interest is associated with engagement across different student populations and institutional settings, with some models explaining substantial variance in engagement outcomes (64, 65). Even in structured and physically demanding activities such as martial arts, engagement has been linked to goal alignment, personal relevance and perceived value—dimensions that correspond to SUV and SAVKSI (66, 67). These patterns are also consistent with ecological perspectives, which emphasize the interaction between individual motivation and contextual conditions in shaping engagement (68, 69).

Taken together, these findings demonstrate the applicability of the tripartite model of individual interest across general, technical and culturally grounded PE contexts. However, despite this growing body of evidence, Filipino recreational games remain unexamined within this framework. This gap is particularly important given the cultural significance and participatory nature of these activities, which may offer unique insights into how interest is associated with engagement in Physical Education.

Although prior research has examined the relationship between PE participation and broader academic outcomes such as performance and well-being (70–72), comparatively fewer studies have focused on the motivational mechanisms operating within PE itself. In particular, limited attention has been given to how culturally grounded movement practices relate to students' psychological engagement. Addressing this gap, the present study investigates the association between individual interest and study engagement in Filipino recreational games-based Physical Education.

### Objectives and hypotheses formulation

This cross-sectional study examined the association between university students' individual interest and their study engagement within the context of Filipino recreational games-based physical education. Situated within the framework of the Tripartite Model of Individual Interest (38), the investigation focuses on how the three core factors are related to students' engagement in this culturally grounded academic subject. Although previous studies in general, cultural and technical PE contexts have shown that individual interest is positively associated with study engagement, this study extends the model into an unexamined but educationally rich domain. Grounded in this framework, it is hypothesized that the three factors of individual interest are positively associated with students' study engagement. Furthermore, it is expected that the overall construct of individual interest, as represented by these dimensions, is likewise positively associated with study engagement in Filipino recreational games-based PE.

## Materials and methods

### Participants

Participants in this study were *purposively* recruited from undergraduate students enrolled in PATH-Fit 4 (Physical Activity Towards Health and Fitness 4), the fourth course in the general physical education sequence commonly implemented in Philippine higher education, during the Second Semester of Academic Year 2024–2025 at a state university in the Philippines. Students were drawn from approximately 10 PATH-Fit 4 sections, with about 40 to 50 students per section, coordinated through approximately 4 to 5 instructors who were requested to disseminate the survey link to classes implementing Filipino recreational games as part of their course content, ensuring that participants had direct exposure to the instructional context examined in this study. These figures are presented as approximate estimates based on the recruitment coordination process, as survey dissemination ultimately depended on voluntary instructor participation and individual student response. A total of 388 questionnaire responses were collected. Following data screening, 27 responses were removed due to incompleteness or lack of informed consent, resulting in a final sample of 361 valid participants and a response rate of 93.0%. An *a priori power analysis* was conducted using G*Power 3.1 (73) for hierarchical multiple regression using the R² increase approach. Results indicated that the required sample size ranged from approximately 119 to 150 participants, depending on the number of predictors entered at each step, assuming a medium effect size (*f*^2^ = 0.15), an alpha level of.05, and 95% statistical power. The final sample of 361 participants exceeded this requirement.

As part of the PATH-Fit 4 course, Filipino recreational games were implemented as a component of the regular physical education instruction during the semester. Classes typically met once per week for approximately two hours, following the standard schedule of the university's general physical education curriculum. Multiple instructors were assigned to handle different PATH-Fit 4 classes, and although instructional approaches may have varied slightly across sections, the core learning activities involved participation in traditional Filipino games such as *patintero, luksong tinik* and *tumbang preso*. These activities were incorporated into the course as part of the movement exploration component, allowing students to actively engage in culturally rooted physical activities throughout the semester.

Of the 361 participants, 146 (40.4%) identified as men, 170 (47.1%) as women and 45 (12.5%) identified as members of the LGBTQIAP + community. In terms of age, the majority were 18 years old (*n* = 197; 54.6%), followed by 19 years old (*n* = 94; 26.0%), 20 years old (*n* = 33; 9.1%), 17 years old (*n* = 17; 4.7%), 21 years old (*n* = 17; 4.7%), and 22 years old (*n* = 3; 0.8%). The mean age was 18.57 years. The demographic distribution of the participants are presented in Table 1.

**Table 1:** Demographic distribution of the participants (*n* = 361).

Demographic profiles	Items	*N* (%)
Sex/gender identity	Men	146 (40.4)
	Women	170 (47.1)
	LGBTQIAP+	45 (12.5)
Age (*x¯* = 18.57)^a^	17 years old	17 (4.7)
	18 years old	197 (54.6)
	19 years old	94 (26.0)
	20 years old	33 (9.1)
	21 years old	17 (4.7)
	22 years old	3 (0.8)

a Mean age.

### Instruments

The data for this study were collected through an online survey administered via Google Forms. Instructors handling PATH-Fit 4 (Physical Activity Towards Health and Fitness 4), specifically those implementing Filipino recreational games as part of the course content, were requested to disseminate the survey link through the university's official Learning Management System (LMS). The data collection period spanned from November to December 2024, following the completion of all course-related practical and theoretical activities to avoid any disruption or academic coercion during instruction.

The survey questionnaire consisted of three major sections. The first section gathered demographic information such as gender identity and age. The second section featured the Students' Individual Interest in Physical Education Questionnaire developed by Roure et al. (38), which is grounded in the tripartite model of individual interest and captures the affective and cognitive components of sustained interest in physical education contexts. This 14-item instrument measures individual interest across three distinct factors: positive affect and willingness to reengage (PAWR), stored-utility value (SUV), and stored attainment value and knowledge-seeking intentions (SAVKSI). Responses were recorded on a 5-point Likert scale ranging from 1 (Strongly Disagree) to 5 (Strongly Agree). In adapting the SIIPEQ to the current study context, minor contextual wording adjustments were made (e.g., replacing general references to “physical education activities” with “Filipino recreational games”) to align the items with the instructional context while preserving the original construct meaning. Given that the modifications were minimal and limited to contextual wording, no formal pilot testing or additional expert validation was conducted. However, the adequacy of the adapted items was supported by satisfactory reliability and validity indicators in the measurement model. The third section of the survey included the Utrecht Work Engagement Scale for Students (UWES-9S) developed by Carmona-Halty et al. (54), which consists of nine items designed to measure students' study engagement across three dimensions: vigor, dedication, and absorption, using a 6-point Likert scale ranging from 0 (Never) to 6 (Always). For the purposes of this study, a composite engagement score was computed to represent students' overall level of study engagement. The questionnaires were administered in English, which is the primary medium of instruction in Philippine higher education.

#### Instrument reliability, validity, and contextual application

To ensure the psychometric soundness of the measurement instruments, confirmatory factor analysis was conducted using PLS-SEM (74). PLS-SEM was selected due to its robustness and its suitability for evaluating measurement models in applied research contexts, particularly when the objective is to assess construct reliability and validity prior to subsequent analyses rather than to test a fully specified covariance-based model. This approach is appropriate for studies that emphasize prediction-oriented and context-specific model evaluation. During the measurement model evaluation, several items with insufficient factor loadings were removed to improve construct reliability and validity. Only items with standardized loadings above the recommended threshold of ≥0.70 were retained in the final model (74). Specifically, five items from the Utrecht Work Engagement Scale for Students (UWES-9S) were removed due to insufficient factor loadings, resulting in a four-item measure (UWES4, UWES5, UWES6 and UWES7). Notably, the retained items represent all three dimensions of the original scale supporting the use of a composite measure of overall study engagement in subsequent analyses.

As presented in Table 2, all retained items exceeded the acceptable threshold, indicating strong indicator reliability. Internal consistency reliability was assessed primarily using composite reliability (CR), which is recommended for PLS-SEM measurement models (74). All constructs demonstrated strong reliability, with CR values ranging from 0.865 to 0.950, exceeding the recommended threshold of ≥0.70. Cronbach's alpha values, reported as a supplementary indicator, also indicated satisfactory reliability (α = 0.863–0.950). Each construct also demonstrated convergent validity, as the average variance extracted (AVE) values were all ≥0.50, satisfying the criterion for construct-level variance capture (74). Furthermore, to evaluate common method bias (CMB), variance inflation factor (VIF) scores were examined using full collinearity assessment. All VIF values fell between 1.975 and 4.456, well below the threshold of <5.00 suggested by Kock (75), indicating that CMB was not a threat to the validity of the results.

**Table 2:** Factor loadings, reliability coefficients, average variance extracted (AVE), and variance inflation factor (VIF) for CMB of study constructs.

Construct	Item	Item loadings	CA	CR	AVE	VIF
PAWR	PAWR1	0.868	0.884	0.887	0.742	2.509
	PAWR2	0.866				2.583
	PAWR4	0.860				2.156
	PAWR5	0.851				2.148
SUV	SUV2	0.858	0.863	0.865	0.785	1.975
	SUV3	0.907				2.580
	SUV4	0.891				2.292
SAVKSI	SAVKSI1	0.886	0.950	0.950	0.832	3.279
	SAVKSI2	0.912				3.871
	SAVKSI3	0.923				4.456
	SAVKSI4	0.918				4.291
	SAVKSI5	0.922				4.391
UWES	UWES4 (DE)	0.865	0.894	0.895	0.759	2.490
	UWES5 (VI)	0.863				2.426
	UWES6 (AB)	0.870				2.532
	UWES7 (DE)	0.886				2.811

Items with factor loadings < 0.70 during the measurement model evaluation were removed prior to the final analysis. Item loadings > 0.70, Cronbach's Alpha (CA) and Composite Reliability (CR) > 0.70, AVE (Average Variance Extracted) > 0.50, VIF (Variance Inflation Factor) < 5.0.

PAWR- Perceived affect and willingness to reengage, SUV- Stored-utility value, SAVKSI- Stored attainment value and knowledge-seeking intentions, UWES-9S- Study engagement, VI- Vigor, DE- Dedication- AB- Absorption.

Lastly, Discriminant validity was assessed using the Fornell-Larcker criterion and the Heterotrait-Monotrait Ratio (HTMT), both reported in Table 3. The square roots of the AVE for each construct exceeded their inter-construct correlations, meeting the Fornell-Larcker condition (74, 76). Additionally, all HTMT values ranged from 0.652–0.842, remaining below the conservative threshold of 0.85, which supports the empirical distinctiveness of the latent constructs (74, 77).

**Table 3:** Fornell-Larcker criterion for discriminant validity among latent constructs and heterotrait-monotrait ratio (HTMT) for discriminant validity among constructs.

Fornell-larcker criterion				
	PAWR	SAVKSI	SUV	UWES
PAWR	0.861			
SAVKSI	0.693	0.912		
SUV	0.737	0.705	0.886	
UWES	0.583	0.658	0.612	0.871
Heterotrait-monotrait r(HTMT)				
PAWR				
SAVKSI	0.752			
SUV	0.842	0.780		
UWES	0.652	0.713	0.696	

HTMT ratio value < 0.85 (conservative) < 0.90 (liberal) approach.

### Data analysis

A two-step analytical approach was employed in this study. This approach was adopted to ensure that the measurement properties of the constructs were established prior to examining their associations, thereby enhancing the validity and interpretability of the subsequent regression results. First, *confirmatory factor analysis* (CFA) using PLS-SEM was conducted to evaluate the reliability and validity of the measurement model prior to hypothesis testing using SmartPLS 4. Second, to examine the association between students' individual interest and their level of study engagement in Filipino recreational games-based physical education, *hierarchical multiple regression analysis* was performed using IBM SPSS Statistics version 29 for macOS. In Model 1, age and gender identity were entered as control variables, with gender identity dummy-coded using men as the reference group. In Model 2, the three factors of individual interest (PAWR, SUV and SAVKSI) were entered as predictor variables, while study engagement served as the criterion variable. This approach was used to assess whether the observed relationships remained consistent after accounting for basic demographic characteristics. Descriptive statistics and bivariate correlations were also computed in SPSS to examine the distributional properties and associations among the study variables. As shown in Table 4, mean (*x¯*) scores ranged from 3.64 to 4.15, with standard deviations between 0.85 and 0.88. All variables exhibited skewness and kurtosis values within ±2.0, indicating acceptable normality. Correlation analysis revealed significant positive associations among all constructs, with SAVKSI showing the strongest correlation with study engagement (*r* = .66, *p* < .01), followed by SUV (*r* = .61, *p* < .01) and PAWR (*r* = .58, *p* < .01).

**Table 4:** Descriptive statistics, distribution properties, and intercorrelations among study variables.

Constructs	$\bar{x}$ ± SD	Skew	Kurt	*1*	*2*	*3*
Age	18.57 ± .95	1.184	1.345			
Gender identity	1.72 ± .67	.400	−.799			
PAWR	3.64 ± .85	−.228	−.386			
SUV	3.80 ± .88	−.417	−.460	.74**		
SAVKSI	4.15 ± .85	−1.017	.810	.69**	.70**	
UWES	3.88 ± .85	−.554	−.197	.58**	.61**	.66**

PAWR, positive affect and willingness to reengage; SUV, stored-utility value; SAVKSI, stored attainment value and knowledge-seeking intentions; UWES, study engagement.

** Significance is at *p* < 0.01 (2-tailed).

### Ethical statement

This research was conducted in accordance with the ethical standards of the Bulacan State University Research Ethics Guidelines. The study protocol (CSER-CRDU-2025-031) was reviewed by the Local Ethics Research Committee of the College of Sports, Exercise and Recreation, and was deemed exempt from full ethical review in accordance with institutional policy, as the study involved minimal-risk survey procedures among university students. All procedures adhered to the provisions of Republic Act No. 10173, also known as the Philippine Data Privacy Act of 2012, ensuring that participants' information and responses were treated with strict confidentiality and anonymity. Participation in the study was entirely voluntary. Prior to completing the questionnaire, respondents were informed about the purpose of the study, the voluntary nature of their participation, and their right to decline or withdraw at any time without academic consequence. Informed consent was obtained from all participants before the survey was administered. Because participants were recruited from regularly enrolled university classes, some students were below 18 years of age at the time of data collection. These individuals participated voluntarily under the same informed consent procedures applied to all respondents in accordance with institutional ethical guidelines for minimal-risk educational research involving enrolled university students.

## Results

**Figure 2:**
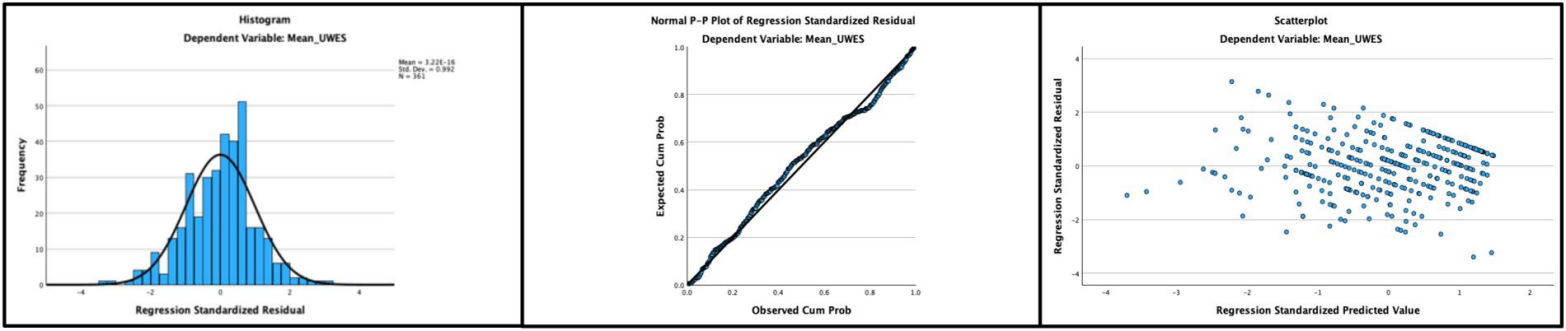
Side-by-side regression diagnostic plots. (Left) Histogram of standardized residuals, (center) normal probability (P–P) plot of standardized residuals, and (right) scatterplot of standardized residuals against standardized predicted values. The plots collectively support the assumptions of normality, linearity and homoscedasticity for the hierarchical multiple regression model associating with study engagement.

Prior to conducting the hierarchical multiple regression analysis, essential assumptions were assessed to ensure the robustness of the results. Visual inspection of the histogram, scatterplot of standardized residuals and the Normal P-P plot of regression standardized residuals (see Figure 2) revealed no violations of normality, linearity or homoscedasticity. The scatterplot showed a roughly random distribution of residuals around zero, supporting the assumptions of linearity and homoscedasticity (78). The points in the Normal P-P plot closely followed the diagonal line, indicating that the residuals were approximately normally distributed (79).

Multicollinearity was also assessed using variance inflation factor (VIF) values (80). All factors showed VIF values below the conservative threshold of <5 (2.278 to 2.648), and tolerance values were above.30, indicating no significant multicollinearity among the factors (81). The Durbin-Watson statistic was 2.118, which is within the acceptable range (1.5–2.5), suggesting no substantial autocorrelation in the residuals (82). These diagnostics confirm that the assumptions of hierarchical multiple regression were adequately met, allowing for valid interpretation of the subsequent analysis.

As can be seen in Table 5, the association between students' individual interest and their study engagement in Filipino recreational games-based physical education was examined using hierarchical multiple regression analysis. In Model 1, age and gender identity were entered as control variables. This model was not statistically significant [*F*(3, 357) = 1.43, *p* = .233], and explained a small proportion of variance in study engagement (*R*^2^ = .012), indicating that the model accounted for approximately 1.2% of the variance. In Model 2, the three factors of individual interest (PAWR, SUV and SAVKSI) were added. The inclusion of these variables resulted in a statistically significant increase in explained variance [Δ*R*^2^ = .475, Δ*F*(3, 354) = 109.22, *p* < .001]. The final model was statistically significant [*F*(6, 354) = 55.98, *p* < .001], and explained 48.7% of the variance in study engagement (*R*^2^ = .487, adjusted *R*^2^ = .478). Furthermore, the effect size of the model was examined using Cohen's *f*^2^ (*f*^2^ = 0.95), indicating a large effect size (83). Additionally, the incremental effect of the individual interest factors was substantial (Δ*f*^2^ = 0.93), further demonstrating the strong practical significance of the model in explaining students' study engagement. At the factor level, SAVKSI showed the strongest practical contribution, followed by SUV and PAWR, consistent with the standardized coefficients.

**Table 5:** Hierarchical regression analysis of the association between individual interest and study engagement.

Variable	Model 1			Model 2			95% CI (LL)	95% CI (UL)
	*B*	*SE B*	*β*	*B*	*SE B*	*β*		
Control variables								
Gender identity (Women vs. men)	−.125	.096	−.073	−.051	.071	−.030	−.190	.088
Gender identity (LGBTQIAP + vs. men)	.005	.145	.002	.042	.105	.016	−.165	.249
Age (in years)	.068	.047	.076	.044	.034	.049	−.024	.111
Individual interest factors								
PAWR	—	—	—	.130	.061	.129*	.009	.251
SUV	—	—	—	.228	.060	.236**	.110	.346
SAVKSI	—	—	—	.400	.058	.398**	.286	.514
Model Fit								
*R*^2^		.012			.487			
Adjusted *R*^2^		.004			.478			
Δ*R*²		—			.475**			
*F* for change in *R*^2^		1.431			109.216**			

B- unstandardized coefficient; SE B- standard error; *β-* standardized coefficient CI- confidence interval. Gender identity was dummy-coded with men as the reference group. Significance is at

* *p* < .05,

** *p* < .001.

PAWR, positive affect and willingness to reengage; SUV, stored-utility value; SAVKSI, stored attainment value and knowledge-seeking intentions.

Because class- or instructor-level identifiers were not recoverable with sufficient confidence, a design effect sensitivity analysis was conducted to evaluate the potential impact of clustering on the inferential results. Using an estimated average cluster size of 36.1 students per section, based on approximately 10 PATH-Fit 4 sections and 361 valid respondents, design effects were calculated under plausible intraclass correlation coefficient (ICC) values of.05 and.10. The resulting design effects were 2.76 and 4.51, corresponding to effective sample sizes of approximately 131 and 80, respectively. Under the more modest clustering assumption (ICC = .05), the principal inferential pattern remained substantively similar, with SAVKSI retaining the strongest association and SUV remaining meaningfully associated with study engagement. Under the more conservative assumption (ICC = .10), the strongest association for SAVKSI remained evident, whereas weaker associations became more sensitive to clustering assumptions. These results suggest that the principal inferential pattern of the study, particularly the prominence of SAVKSI, remains reasonably robust, although smaller effects should be interpreted with greater caution.

In the final model, SAVKSI emerged as the strongest factor of study engagement (*β* = .40, *t*(354) = 6.92, *p* < .001, 95% CI [0.29, 0.51). This factor reflects students' internalized valuation of physical education as meaningful for personal growth and knowledge development. In the context of Filipino recreational games, this finding underscores the importance of embedding cultural identity and deeper meaning into instructional practices to support sustained engagement. Similarly, SUV was significantly associated with study engagement (*β* = .24, *t*(354) = 3.81, *p* < .001, 95% CI [0.11, 0.35). This reflects students' perceived usefulness of physical education, particularly in relation to health and fitness outcomes. The significance of SUV highlights the utilitarian dimension of individual interest, suggesting that when students perceive functional or long-term benefits, higher engagement is observed. PAWR was also significantly associated with study engagement (*β* = .13, *t*(354) = 2.11, *p* = .035, 95% CI [0.01, 0.25]. Although the weakest among the factors, PAWR reflects students' emotional resonance and willingness to reengage based on prior enjoyment, suggesting that affective experiences serve as important motivational entry points in participation.

## Discussion

The present study provides further support for the role of individual interest as a significant construct associated with study engagement in Filipino recreational games-based physical education. Anchored in Roure et al. (38) tripartite model, the findings further affirm that when students experience physical education through the lenses of affective resonance, perceived utility, and personal relevance, higher levels of engagement tend to be observed. This aligns with Self-Determination Theory (38), which posits that intrinsic motivational resources such as enjoyment, perceived competence, and value attribution are central to sustained academic engagement. Likewise, Expectancy-Value Theory (40) supports this perspective, emphasizing that learners are more likely to invest effort in activities they both value and believe they can successfully perform.

Importantly, the associations observed in this study remained consistent even after controlling for basic demographic variables such as age and gender identity. This suggests that the relationship between individual interest and study engagement is robust across these characteristics, reinforcing the role of interest as a key motivational construct in physical education contexts. The stability of these findings provides additional confidence that the observed relationships are not merely attributable to demographic differences but reflect meaningful psychological processes underlying student engagement.

The consistency of these findings with prior studies conducted in other physical education contexts suggests that the tripartite model of individual interest may demonstrate stable associations with study engagement across diverse movement domains. Rather than indicating redundancy, this pattern provides evidence of the model's cross-context robustness, particularly within culturally grounded physical education settings. In this regard, the present study contributes to a growing programmatic line of research that examines how individual interest operates across varied instructional contexts, including both structured and play-based forms of physical activity.

A more explicit comparison with prior studies further clarifies this cross-context pattern. In the present study, the standardized coefficients showed that SAVKSI had the strongest association with study engagement (*β* = .398), followed by SUV (*β* = .236) and PAWR (*β* = .129). This ordering broadly converges with prior findings in general PE, where SAVKSI also emerged as the strongest dimension in both the large multi-institution study (PAWR = .157, SUV = .164, SAVKSI = .444) and the two-college study (PAWR = .097, SUV = .268, SAVKSI = .326). A similar pattern was also evident in Philippine folk dance, where SAVKSI was clearly dominant (PAWR = −.015, SUV = .123, SAVKSI = .483). By contrast, gymnastics displayed a different configuration, with SUV emerging as the strongest coefficient (PAWR = −.096, SUV = .307, SAVKSI = .107), suggesting that perceived usefulness may become more salient in technical or performance-oriented activities. Taken together, these comparisons suggest that SAVKSI may be the most stable dimension across general and culturally grounded PE contexts, whereas PAWR and SUV appear more context-sensitive depending on the nature of the movement activity.

Among the three factors, SAVKSI showed the strongest association with study engagement. This result resonates with studies emphasizing the role of meaningfulness and personal identity in academic motivation (84, 85). Students who recognize physical education as a domain that nurtures cultural understanding, personal growth, or even professional aspirations tend to demonstrate deeper and more enduring engagement (44). Particularly in a Filipino context, where recreational games are not only playful activities but also conduits of national identity and heritage (21, 24), this connection reinforces the necessity of culturally sustaining pedagogy in movement education (86). It also highlights how physical education can move beyond bodily engagement to become a platform for civic awareness and reflective learning (87).

Meanwhile, the association of SUV with study engagement illustrates the utilitarian dimension of individual interest (88). This finding supports prior research suggesting that when students perceive physical education as beneficial to their health, social development, or academic functioning, higher levels of engagement tend to be observed (96). Filipino recreational games, traditionally framed as leisure-oriented activities, are thus repositioned as functional learning experiences (27), offering opportunities to develop endurance, coordination, teamwork, and resilience. The significance of SUV reinforces the view that educators must intentionally connect physical education content with real-life applications to underscore its relevance and long-term value (89).

Although PAWR showed the weakest association among the three factors, it still demonstrated a statistically meaningful relationship with study engagement. This supports the notion that enjoyment and emotional connection function as initial motivational triggers (90), particularly in physical activity contexts (38). While affective engagement alone may not sustain long-term involvement (91), it plays a critical entry role in fostering participation. For instance, the social and nostalgic appeal of *laro ng lahi* may attract students who might otherwise disengage from more structured or competitive forms of physical education. Thus, fostering positive emotional experiences should not be viewed as an endpoint, but rather as a gateway toward deeper and more enduring forms of interest.

Despite the substantial variance accounted for in the model, a considerable portion of students' engagement remains unexplained. This suggests that other factors, such as perceived autonomy, instructor support, or contextual conditions (e.g., class scheduling, physical environment), may also play important roles (92). Prior research has shown that environmental and social dynamics within physical education settings significantly shape student motivation (93). In this regard, broader theoretical perspectives such as Bronfenbrenner's ecological systems theory (68, 69) and the person-environment (P-E) fit model (94) offer valuable lenses for examining how multiple layers of influence interact to shape engagement in movement-based learning.

The findings of this study contribute to the growing body of Philippine-based research on motivation in movement education, where empirical investigations remain relatively limited. Previous studies in the local context have often focused on general academic engagement or narrowly defined constructs such as physical activity preferences. In contrast, the present study advances a more nuanced and theory-driven understanding of how culturally grounded physical education practices intersect with motivational processes. By empirically supporting the tripartite model within a recreational game context, the study extends the applicability of interest-based frameworks in physical education and highlights the value of integrating localized cultural content with universal motivational theories.

Ultimately, the findings suggest that educational stakeholders and curriculum designers should not only preserve Filipino recreational games as cultural heritage practices but also strategically position them as pedagogical tools for fostering sustained interest in physical activity. This may require a shift in instructional orientation—from content coverage toward meaning-making, and from performance metrics toward identity formation and personal relevance. In a time when many young individuals are increasingly disengaged from movement, embedding cultural, emotional and functional significance into physical education may serve as a critical pathway toward rekindling interest and deepening engagement.

## Conclusion

This study examined the association between individual interest and study engagement in Filipino recreational games-based PE. The findings indicate that all three factors were positively associated with students' engagement levels, with SAVKSI showing the strongest association, followed by SUV and PAWR. These results highlight that individual interest is not a monolithic experience but a multidimensional construct shaped by emotional enjoyment, perceived usefulness and deeper personal relevance. Importantly, these associations remained consistent even after accounting for basic demographic characteristics, suggesting that the relationship between individual interest and study engagement is robust across age and gender identity. This reinforces the role of interest as a meaningful motivational construct within physical education contexts.

Crucially, Filipino recreational games appear to provide a context that supports meaningful engagement when approached with pedagogical intentionality. Rather than treating these games as mere heritage artifacts or informal play, they may be positioned as purposeful educational tools when aligned with students' internal motivations. By supporting the applicability of the tripartite model within this culturally rooted context, the study underscores that traditional movement practices can be associated with academically meaningful engagement when they connect with learners' psychological needs.

### Implications

The findings carry meaningful implications for physical education practice and curriculum design in higher education. First, instructors should adopt interest-based pedagogies that integrate emotionally positive experiences, demonstrate real-life relevance, and help students construct personal meaning. Educators can structure PE courses to go beyond physical performance and foster intellectual and emotional connections to content. Especially in heritage-based games, which naturally lend themselves to discussions of culture, identity, and socialization. Additionally, curriculum planners should consider embedding Filipino recreational games within general PE programs not just for cultural preservation, but as a vehicle to increase engagement and motivation. When students perceive that these games contribute to personal development and community belonging, they are more likely to invest effort and sustain participation.

From a practical standpoint, implementing Filipino recreational games in higher education physical education courses may also require institutional and instructional considerations. Faculty development programs may incorporate short workshops or training sessions that equip instructors with strategies for integrating traditional games into structured lesson plans while maintaining clear learning outcomes. Because many Filipino recreational games require minimal equipment and adaptable spaces, they can be effectively implemented even in classes with limited facilities or large enrollments. Instructors may structure activities through rotating small-group formats to accommodate large class sizes while maintaining active participation. Allocating dedicated instructional time within the PATH-Fit curriculum for culturally grounded movement activities may further help sustain student interest and engagement while reinforcing the cultural relevance of physical education.

### Limitations

Despite yielding insightful findings, this study is not without limitations. First, the sample was drawn exclusively from a state university in the Philippines. Although this context provides valuable insight into a localized physical education setting, the single-institution scope limits the generalizability of the results across diverse educational environments both within and beyond the country. Second, the cross-sectional design restricts the ability to draw causal inferences between individual interest and study engagement. Although significant associations were identified, longitudinal or experimental designs are needed to better understand the temporal and directional dynamics between these variables. Third, data were collected through self-report questionnaires, which may be subject to response biases such as social desirability or inflated perceptions of engagement. Although common method bias (CMB) was assessed using full collinearity diagnostics and variance inflation factor values were found to be within acceptable thresholds, the possibility of residual method bias cannot be entirely ruled out given that all variables were measured at a single point in time. Fourth, the reduction of the Utrecht Work Engagement Scale for Students (UWES-9S) to four items, although still representing all three engagement dimensions, may limit the full capture of its original multidimensional structure and the nuance of the engagement construct. Fifth, participants were drawn from multiple physical education classes handled by different instructors; however, class- or instructor-level identifiers were not collected. As a result, it was not possible to directly estimate intraclass correlation coefficients or model class-level variability using observed grouping data. This represents a methodological limitation, as instructor practices and class-level dynamics may influence both individual interest and study engagement. To partly address this concern, a design effect sensitivity analysis using plausible ICC values was conducted, suggesting that the principal inferential pattern remained reasonably stable, although smaller associations should be interpreted with greater caution. Nevertheless, the data were collected through voluntary individual responses rather than structured sampling within intact class groups, which may reduce the likelihood of strong clustering effects. Future research is encouraged to incorporate class- or instructor-level identifiers to enable multilevel modeling or clustering diagnostics, thereby providing a more comprehensive understanding of contextual influences in physical education settings. Finally, although Filipino recreational games provide a culturally rich and contextually meaningful platform for engagement, the findings may not readily generalize to other physical education content areas such as dance, sports, or fitness-based activities without further empirical validation.

### Future research directions

Future studies are encouraged to examine how the tripartite model of individual interest operates across different physical education content areas and educational levels. Comparative investigations involving dance, competitive sports or outdoor adventure education (OAE) may provide insight into whether similar motivational patterns are observed across varied movement contexts, and whether the relative strength of the individual interest dimensions differs across these settings. Moreover, the use of qualitative methodologies, such as phenomenological interviews or classroom observations, may offer deeper insight into how individual interest is experienced, expressed and sustained within authentic learning environments. These approaches can complement quantitative findings by capturing the nuanced and evolving nature of student engagement.

Future research should also consider incorporating class- or instructor-level identifiers to enable the examination of potential clustering effects. The use of intraclass correlation coefficients (ICC) and multilevel modeling techniques would allow researchers to account for nested data structures and better understand how instructor practices and class-level dynamics shape both individual interest and study engagement. This is particularly important in physical education contexts, where teaching styles, classroom climate and peer interactions may meaningfully influence student experiences. Additionally, external factors such as instructor behavior, peer dynamics and physical learning environments warrant further investigation, as these may interact with individual interest to shape engagement outcomes. Drawing from ecological perspectives, future models may integrate social, institutional, and environmental variables to provide a more comprehensive understanding of engagement in physical education settings.

Succeeding line of inquiries may also benefit from conducting moderation or comparative analyses (e.g., across gender, prior experience or different physical education contexts) to examine whether the relative influence of the individual interest dimensions varies under different conditions. This direction would extend the model beyond cross-context consistency toward identifying meaningful contextual differences and strengthening its theoretical contribution. Given that the current model accounted for approximately 48% of the variance in study engagement, future research may benefit from incorporating additional predictors to enhance explanatory power and further refine interest-based frameworks in movement education.

### Contributions to the global discourse

This study adds to the growing global conversation on culturally responsive physical education and the psychology of student engagement. It challenges the often Western-centric PE literature by presenting empirical evidence from an underrepresented cultural framework. In doing so, it expands the applicability of motivational theories like the tripartite interest model and reinforces the idea that educational value lies not only in physical skill acquisition but also in cultural affirmation and personal meaning-making. By elevating Filipino recreational games within academic inquiry, the study advocates for a decolonized approach to physical education. One where local movement traditions are not romanticized nor relegated, but systematically studied and pedagogically leveraged to build more inclusive, motivating and enriching educational experiences.

## Data Availability

The original contributions presented in the study are included in the article/Supplementary Material, further inquiries can be directed to the corresponding author.
